# Rad51 and BRCA2 - New Molecular Targets for Sensitizing Glioma Cells to Alkylating Anticancer Drugs

**DOI:** 10.1371/journal.pone.0027183

**Published:** 2011-11-02

**Authors:** Steve Quiros, Wynand Paul Roos, Bernd Kaina

**Affiliations:** Institute for Toxicology, Medical Center of the Johannes Gutenberg University Mainz, Mainz, Germany; IIT Research Institute, United States of America

## Abstract

First line chemotherapeutics for brain tumors (malignant gliomas) are alkylating agents such as temozolomide and nimustine. Despite growing knowledge of how these agents work, patients suffering from this malignancy still face a dismal prognosis. Alkylating agents target DNA, forming the killing lesion O^6^-alkylguanine, which is converted into DNA double-strand breaks (DSBs) that trigger apoptosis. Here we assessed whether inhibiting repair of DSBs by homologous recombination (HR) or non-homologous end joining (NHEJ) is a reasonable strategy for sensitizing glioma cells to alkylating agents. For down-regulation of HR in glioma cells, we used an interference RNA (iRNA) approach targeting Rad51 and BRCA2, and for NHEJ we employed the DNA-PK inhibitor NU7026. We also assessed whether inhibition of poly(ADP)ribosyltransferase (PARP) by olaparib would enhance the killing effect. The data show that knockdown of Rad51 or BRCA2 greatly sensitizes cells to DSBs and the induction of cell death following temozolomide and nimustine (ACNU). It did not sensitize to ionizing radiation (IR). The expression of O^6^-methylguanine-DNA methyltransferase (MGMT) abolished all these effects, indicating that O^6^-alkylguanine induced by these drugs is the primary lesion responsible for the formation of DSBs and increased sensitivity of glioma cells following knockdown of Rad51 and BRCA2. Inhibition of DNA-PK only slightly sensitized to temozolomide whereas a significant effect was observed with IR. A triple strategy including siRNA and the PARP inhibitor olaparib further improved the killing effect of temozolomide. The data provides evidence that down-regulation of Rad51 or BRCA2 is a reasonable strategy for sensitizing glioma cells to killing by O^6^-alkylating anti-cancer drugs. The data also provide proof of principle that a triple strategy involving down-regulation of HR, PARP inhibition and MGMT depletion may greatly enhance the therapeutic effect of temozolomide.

## Introduction

Glioblastoma multiforme (GBM, WHO grade IV) is the deadliest form of malignant brain tumors. Complete surgical resection of this tumor is hampered by its inherent invasiveness into the surrounding healthy brain tissue. As the tumor cannot be removed completely, adjuvant chemo-radiotherapy plays a major role in the treatment of patients. Nevertheless, despite the best available therapeutic approach, the survival rate for patients with this malignancy is below 1.5 years after diagnosis [1]. Temozolomide (TMZ) is the current first-line chemotherapeutic for gliomas. Similar to other methylating chemotherapeutics such as procarbazine, dacarbazine and streptozotocine, TMZ-induced cell-kill is mainly due to O^6^-methylguanine (O^6^MeG), which is a minor lesion induced by these agents in DNA. The suicide enzyme O^6^-methylguanine-DNA methyltransferase (MGMT) repairs O^6^MeG and thereby renders cells resistant to methylating agents [2]. Consequently, MGMT activity and the promoter methylation status of the *MGMT* gene (indicators of O^6^MeG repair capacity and MGMT protein expression, respectively) are used as predictive markers for the response of gliomas to TMZ [3], [4]. As a result of the protective role of MGMT in methylating and chloroethylating agent based therapy, MGMT inhibitors are in trials for use in MGMT expressing tumors [5].

O^6^MeG is processed into DNA double-strand breaks (DSBs) in a DNA mismatch repair (MMR)-dependent manner, which requires two rounds of DNA replication [6], [7], [8], [9]. These DSBs then trigger apoptotic cell death in gliomas [10]. For chloroethylating agents such as nimustine (ACNU), O6-chloroethylguanine forms secondary interstrand crosslinks that in turn give rise to DSBs (for review see [2]). Cells can protect against DSBs via two repair pathways, namely non-homologous end joining (NHEJ) and homologous recombination (HR).

NHEJ is a error-prone process that relies on the coordinated action of Ku70/Ku80, DNA-PKcs, Artemis, XRCC4 and DNA ligase IV, among other factors, to rejoin the two ends of a broken DNA molecule [11], [12]. HR employs sequence homology to perform an error-free break correction that preserves the original DNA sequence. The central reaction of the HR pathway, namely the homology search and strand invasion, is performed by Rad51-coated 3′- single stranded DNA (ssDNA) tails generated by DNA end resection of the break [13], [14]. The formation of this nucleoprotein filament at ssDNA is promoted and stabilized by BRCA2 [15], [16]. Both Rad51 and BRCA2 are essential for HR in mammalian cells. A significant function of Rad51 and BRCA2 in other repair pathways has not been described.

Working with rodent cells mutated in these DSB repair pathways, we were able to show that HR, but not NHEJ, is responsible for resistance to DSBs formed in response to TMZ-induced O^6^MeG [17]. Similar data were obtained for cells treated with chloroethylating agents like ACNU and lomustine (CCNU), which are also used in glioma therapy (Nikolova et al., unpublished data). These findings suggest that HR might be a promising target in methylating and chloroethylating agent based glioma therapy.

This work was aimed at assessing whether knockdown of HR in glioma cells by interference RNA (iRNA) leads to improved killing of glioma cells following treatment with TMZ, ACNU and ionizing radiation (IR), all part of the standard care for patients with this malignancy.

## Results and Discussion

### Knockdown of the homologous recombination proteins Rad51 and BRCA2 sensitizes glioma cells to alkylating anti-cancer drugs

**Figure 1:**
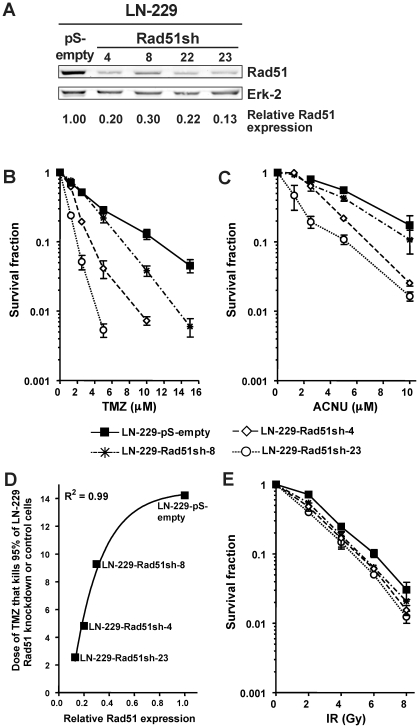
Knockdown of the HR protein Rad51 sensitizes glioma cells to O^6^AA chemotherapeutics. (**A**) Rad51 protein expression in knockdown clones and control assessed by western blot, quantified, corrected for loading (Erk-2) and expressed relative to control. (**B**) Clonogenic survival for stable Rad51 knockdown glioma cells (LN229-Rad51sh) compared to empty vector transfected cells (LN-229-pS-empty) following TMZ treatment. (**C**) Clonogenic survival for stable Rad51 knockdown glioma cells compared to empty vector transfected cells following ACNU treatment. (**D**) Correlation between the relative Rad51 expression, determined from A, and TMZ concentration that kills 95% of glioma cells, determined from B. Line was fitted using the equation y = minimum + (maximum-minimum)×(1-exp(-kx)). (**E**) Clonogenic survival for stable Rad51 knockdown glioma cells compared to empty vector transfected cells following ionizing radiation (IR).

O^6^-alkylating agents (O^6^AA) are widely used as standalone agents or as part of regimes for the treatment of glioma, malignant melanoma, Hodgkin's and non-Hodgkin lymphoma, sarcoma and islet cell carcinoma of the pancreas. To determine whether down-regulation of HR would lead to increased sensitivity of glioma cells to O^6^AA, stable and transient iRNA transfections targeting HR proteins were performed in these cells. LN-229 glioma cells stably knocked-down for Rad51 (Fig. 1A demonstrating parental cells and four stable Rad51sh transfectants), which is the protein responsible for homology search and strand invasion, showed a profound increase in both TMZ (Fig. 1B) and ACNU (Fig. 1C) induced cell death. Remarkably, this increase in sensitivity was proportional to the increase in Rad51 knockdown (Fig. 1D; a similar quantitative correlation was found for ACNU (not shown)). One may suppose that knockdown of Rad51 causes a general sensitization that pertains to all genotoxins including IR. To determine whether this is the case, LN-229 Rad51 knockdown cells were exposed to increasing doses of γ-radiation, which contrary to O^6^AA induce DSBs directly. A slight increase in sensitivity in the Rad51 knockdown lines compared to the control line was observed (Fig. 1E), although not as dramatic as the sensitization towards O^6^AA (compare with Fig. 1B and 1C).

**Figure 2:**
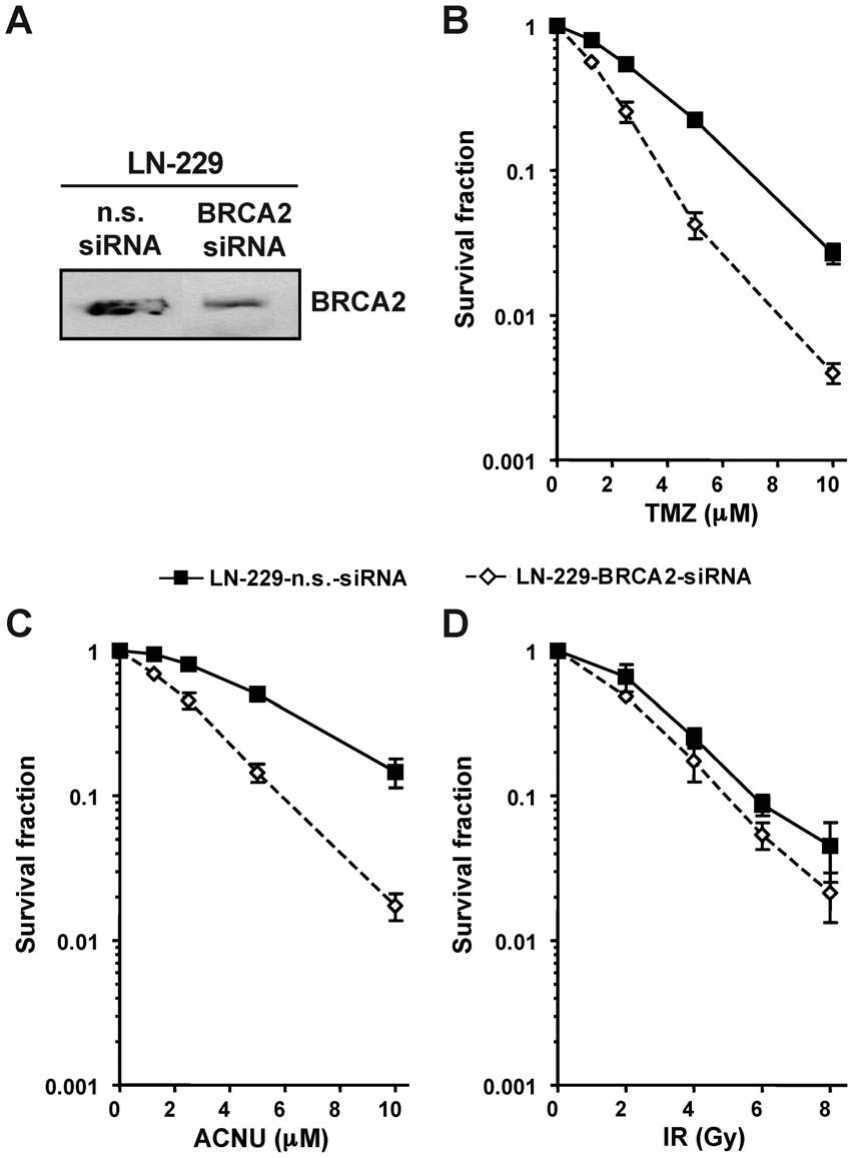
Knockdown of the HR protein BRCA2 sensitizes glioma cells to O^6^AA chemotherapeutics. (**A**) BRCA2 protein expression following knockdown was assessed by western blot. (**B**) Clonogenic survival following TMZ treatments in transient BRCA2 or non-sense (n.s.) siRNA transfected glioma cells. (**C**) Clonogenic survival following ACNU treatments in transient BRCA2 or non-sense (n.s.) siRNA transfected glioma cells. (**D**) Clonogenic survival following ionizing radiation (IR) in transient BRCA2 or non-sense (n.s.) siRNA transfected glioma cells.

To determine whether the sensitization of glioma cells to O^6^AA chemotherapeutics is not exclusive for Rad51 knockdown, BRCA2, the scaffold protein that promotes and stabilizes the presynaptic filament formation during HR repair, was transiently down-regulated (Fig. 2A). Similar to Rad51, BRCA2 knockdown also caused sensitization of glioma cells to TMZ and ACNU (Fig. 2B and 2C). Again, for ionizing radiation the increase in cell kill after BRCA2 knockdown was less prominent (Fig. 2D). These results suggest that HR plays a protective role in glioma cells treated with TMZ or ACNU and that saturation of HR must occur before O^6^AA chemotherapeutics trigger cell death.

**Figure 3:**
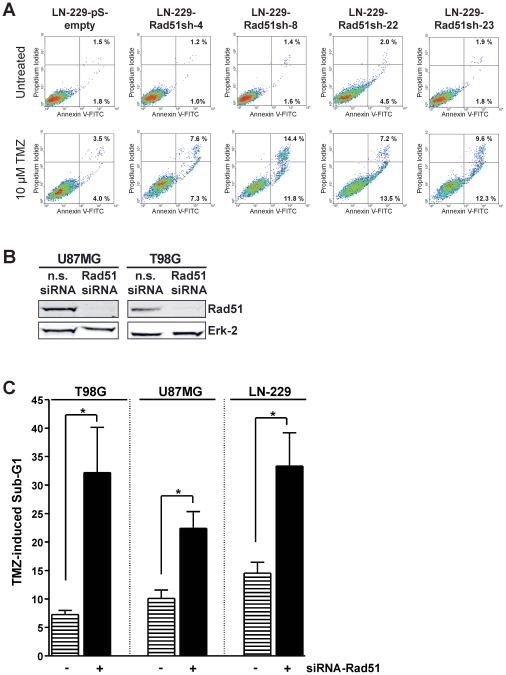
Apoptosis induction after TMZ treatment in Rad51 kd glioma cells. (**A**) Apoptosis determined by annexin V/PI double-staining 144 h after 10 µM TMZ in stable Rad51 knockdown clones of the cell line LN229. (**B**) Western blot analysis of transient Rad51 knockdown in U87MG and T98G cells, or transfected with non-sense siRNA. Erk-2 was used as loading control. (**C**) Apoptosis induction after TMZ treatment in Rad51 transient knocked-down glioma cells U87MG and T98G, as well as in the stable Rad51 knockdown clone LN-229-Rad51-sh8 and the empty vector control cell line LN-229-pS-empty. Apoptosis was assessed by Sub-G1 analysis performed 144 h after 10 µM TMZ treatment. For MGMT depletion 10 µM O^6^BG was added 1 h before TMZ. * p<0.05, significance level determined using the t-student test (n = 3).

Cell death in stable Rad51 down-regulated cells showed a strong increase in apoptosis following TMZ treatment compared to control as determined by annexin V/PI flow cytometry (Fig. 3A). This is in line with previous data obtained with glioma cells showing that apoptosis is the major cell death pathway following TMZ [10]. If Rad51 represents a general mechanism for TMZ resistance in glioma cells, then knockdown of Rad51 in other glioma cells should also lead to increased sensitivity towards TMZ triggered apoptosis. To prove this, Rad51 was transiently knocked-down in the glioma cell lines U87MG and T98G (Fig. 3B). TMZ induced apoptosis was determined in these cells and compared to the response of LN-229 cells transfected with the pS-empty and Rad51sh vector (Fig. 3C). We should note that for the cell line T98G, MGMT was depleted by O^6^BG, while LN-229 and U87MG does not express MGMT and therefore O^6^BG pretreatment was not necessary. All three cell lines showed a significant increase in TMZ triggered apoptosis following Rad51 knockdown (Fig. 3C). These results support the view that the chemo-sensitizing effect of HR down-regulation is a general phenomenon that is not restricted to a particular glioma cell line.

### Sensitization of Rad51 knockdown depends on O^6^-alkylguanine lesions

**Figure 4:**
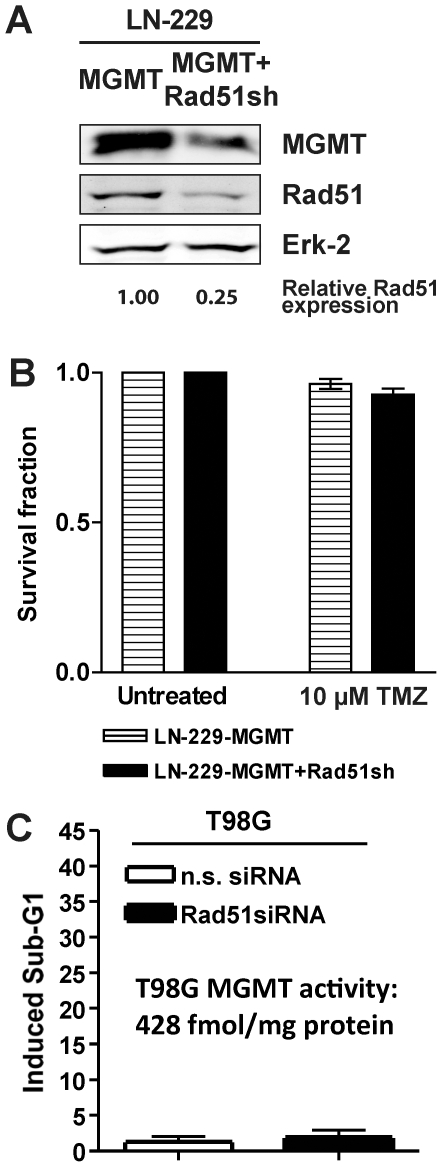
HR downregulation sensitizes to O^6^-alkylguanine lesions in glioma cells. (**A**) Western blot analysis of MGMT and Rad51 protein levels of glioma cells stably transfected to express MGMT (LN-229-MGMT) and cells stably expressing MGMT and knockdown for Rad51 (LN-229-MGMT+Rad51sh). Erk-2 was used for loading control. (**B**) Clonogenic survival for cells stably transfected to express MGMT (LN-229-MGMT) and cells stably expressing MGMT and knockdown for Rad51 (LN-229-MGMT+Rad51sh) after TMZ treatment. (**C**) Apoptosis in T98G glioma cells that were transiently knocked-down for Rad51 (or transfected with non-sense siRNA) treated with TMZ. Apoptosis was determined by Sub-G1 flow cytometry 144 h after TMZ addition.

TMZ methylates DNA at 13 positions [18]. Prominent alkylation lesions are N7-methylguanine, N3-methyladenine, N3-methylguanine and O^6^-methylguanine. In rodent cells, HR has been reported to be the major downstream protection mechanism against both N-alkylations in purines [19] and O^6^-alkylations in guanine [17]. The TMZ-induced adduct O^6^MeG is repaired by MGMT [2]; consequently, over-expression of MGMT in HR knockdown glioma cells should render them resistant to TMZ if HR knockdown sensitizes towards the O^6^MeG adduct. LN-229 does not express MGMT detectably. Therefore, we transfected MGMT into these cells generating stable MGMT transfectants (Fig. 4A). As expected, these cells were highly protected against TMZ-induced cell death. Knockdown of Rad51 in these cells had no significant effect on TMZ-induced toxicity (Fig. 4B, compare with Fig. 1B). The data shows that blocking the HR repair pathway by Rad51 knockdown sensitizes to cell death triggered by the DNA adduct O^6^MeG.

Although brain tumors usually express low levels of MGMT [20], a major fraction of these malignancies do indeed show clinically relevant expression levels of this repair enzyme, making them unsuitable for treatment with O^6^AA. To overcome this problem, MGMT inhibitors are being tested in trials for enhancing the effectiveness of TMZ and related agents. Employing a model that resembles this situation, T98G glioma cells that express endogenous MGMT were knocked-down for Rad51. The MGMT expressing T98G cells were completely resistant to TMZ, irrespective of Rad51 knockdown (Fig. 4C). This data are similar to those obtained with LN-229 MGMT transfected cells, supporting the notion that HR knockdown sensitizes glioma cells to O^6^MeG adducts and that MGMT inhibition greatly improves on this.

### Knockdown of Rad51 in glioma cells prevents the repair of DSBs formed during the processing of O^6^-methylguanine

**Figure 5:**
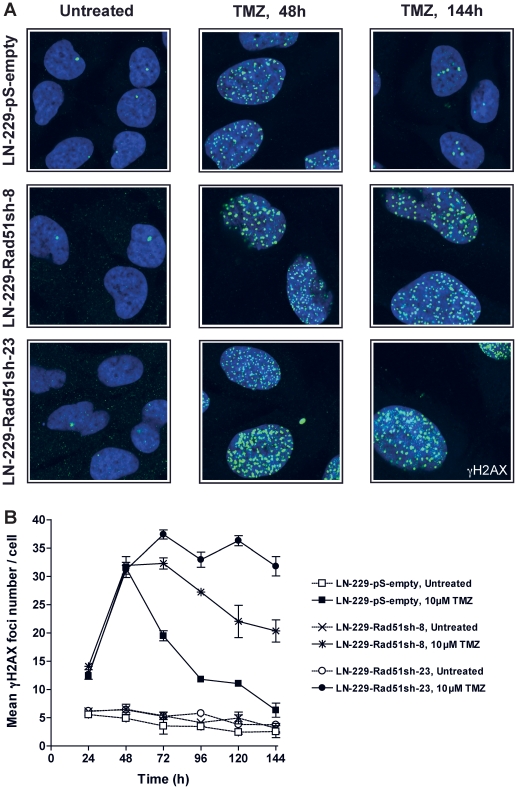
HR down-regulation impairs the repair of TMZ induced DNA-DSBs. Kinetics of TMZ-induced γH2AX foci formation and disappearance in Rad51 knockdown glioma cells treated with 10 µM TMZ. See (**A**) for representative micrographs and (**B**) for quantification. Each measure point represents the mean of 200 cells per experiment. Experiments were repeated twice and mean values +/− SD are shown.

**Figure 6:**
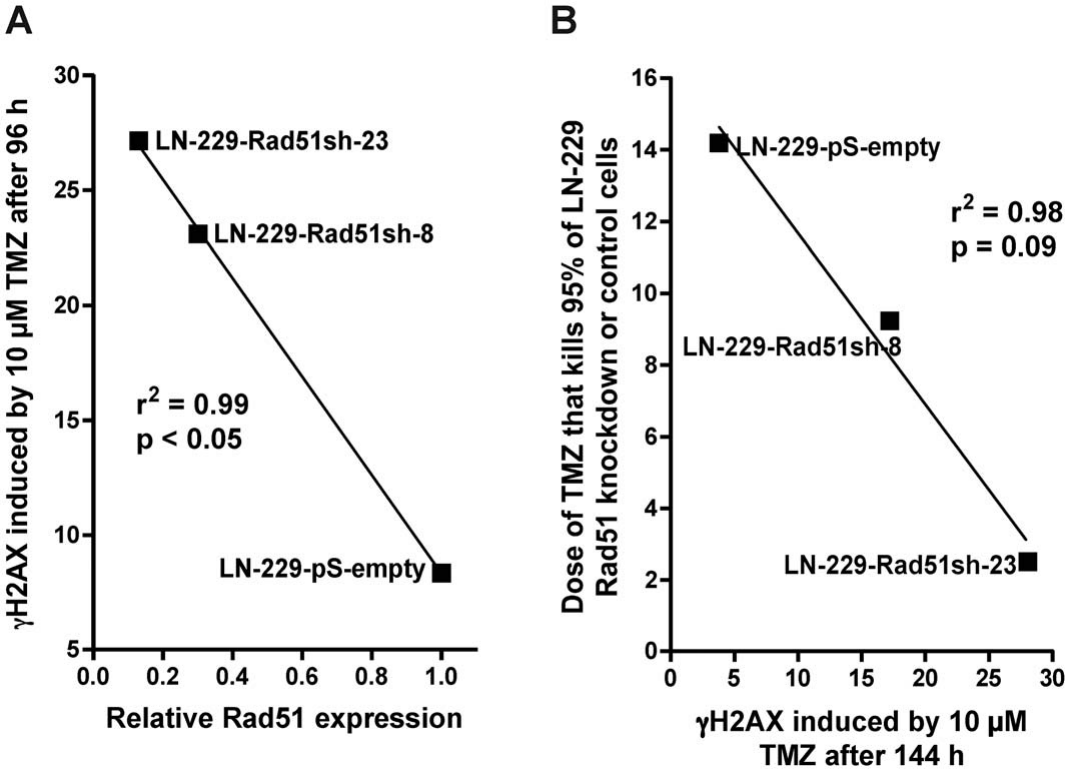
Correlations between Rad51 expression level and γH2AX foci (A) and the amount of γH2AX foci and cell death (B). Relative Rad51 expression was determined from Fig.1A and the doses required to kill 95% of glioma Rad51 knockdown cell lines cells are from Fig.1B.

**Figure 7:**
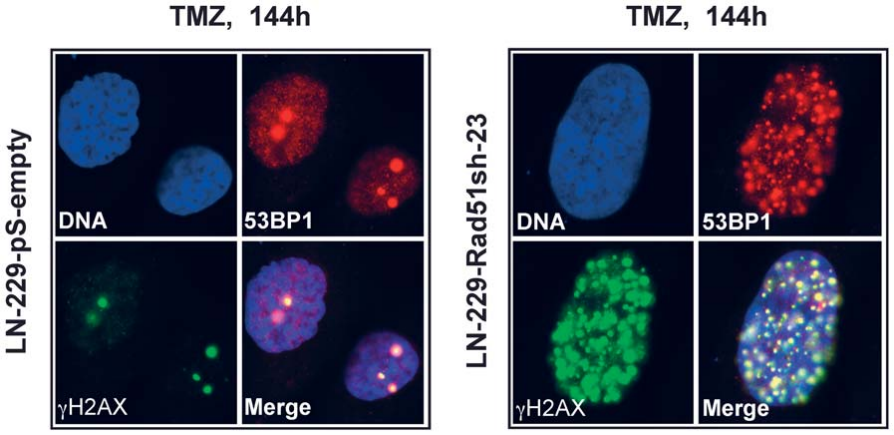
Co-localization of 53BP1 and γH2AX. 53BP1 was immuno-detected as a second marker for DSB. Representative micrographs are shown.

Having shown that knockdown of HR proteins sensitizes glioma cells to O^6^AAs, the question of whether it would also have an effect on the formation and repair of DSBs following TMZ treatment was addressed. A reliable and robust marker for DSBs are phosphorylated histone 2AX (γH2AX) foci [21]. Glioma cells knocked-down in Rad51 were treated with TMZ and compared with non-knockdown cells as to DSB formation and repair (Fig. 5A for representative immunofluorescence and Fig. 5B for quantification). In control cells transfected with the empty vector, DSBs were formed 48 h after treatment and then repaired (Fig. 5B), which conforms to previous data [17]. Contrary to this, in Rad51 knockdown lines (clones 8 and 23) DSBs were formed and remained unrepaired up to 144 h after TMZ treatment (Fig. 5B). Clearly, knockdown of Rad51 in glioma cells caused a significant defect in the repair of DSBs induced by TMZ. The inhibition of DSB repair as determined by γH2AX showed an inverse and significant correlation with Rad51 expression in the knockdown cells (Fig. 6A). The γH2AX foci level that remained after 144 h was also related to cell survival following TMZ (Fig. 6B). To confirm that TMZ-induced γH2AX foci are “true” DSBs, a second DSB marker was used that is known to physically interact with γH2AX, namely 53BP1 [22]. Similar to γH2AX, TMZ also induced the formation of 53BP1 foci, which co-localized with γH2AX (Fig. 7). The data support the notion that Rad51 dependent HR is required for the repair of TMZ-induced DSBs.

### Influence of inhibiting NHEJ or poly(ADP-ribose)polymerase on TMZ treated glioma cells

**Figure 8:**
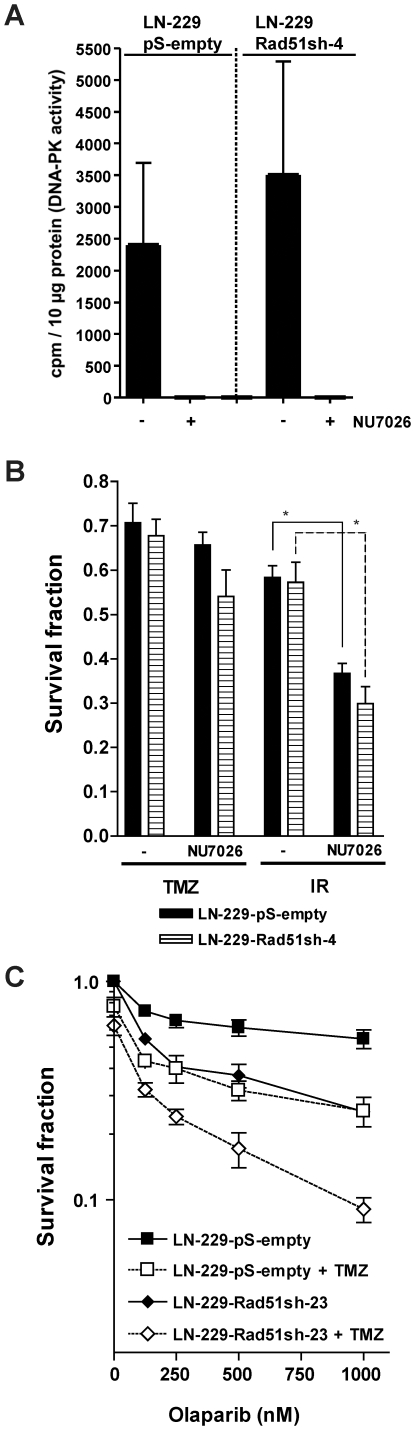
Role of DNA-PK dependent NHEJ and PARP dependent BER on sensitivity of glioma cells knockdown for Rad51 towards TMZ. (**A**) Inhibition of DNA-PK activity with NU7026. DNA-PK activity was determined in cell extracts in the presence and absence of NU7026. (**B**) Clonogenic survival of stable Rad51 knocked-down glioma cells (LN-229-Rad51sh-4) compared to empty vector-transfected cells (LN-229-pS-empty) after TMZ or ionizing radiation (IR) treatment in the presence or absence of the DNA-PKcs inhibitor NU7026. Equitoxic TMZ and ionizing radiation (IR) doses producing about 30 to 40% toxicity in the absence of DNA-PK inhibition were used [TMZ/IR doses: LN-229-pS-empty: 2.7 µM/2.7Gy; LN-229-Rad51sh-4: 1.5 µM/1.9 Gy]. *, p<0.05, significance levels were calculated using the Mann-Whitney U test (n = 5). (**C**) Clonogenic survival of control (LN-229-pS-empty-2) and Rad51 knockdown (LN-229-Rad51sh-23) glioma cells as a function of olaparib concentration. Cells were co-treated with TMZ or not. TMZ doses producing 30 to 40% toxicity in the absence of PARP inhibition were selected for LN-229-Rad51sh-23 (0.8 µM) and control cells (2.5 µM).

As NHEJ may theoretically also play a protective role for methylating agents, independent of HR, its effect was investigated in TMZ-treated glioma cells. A main player in NHEJ is DNA-PKcs, which is a PI3-kinase that binds, together with Ku70 and Ku80, to free DSB ends and stimulates their rejoining and ligation [11], [12]. For inhibiting DNA-PK we used the small molecule inhibitor NU7026 [23], which strongly inhibited DNA-PK activity upon treatment of both LN-229 control (transfected with the empty vector) and LN-229 Rad51 transfected cells (Fig. 8A). Inhibition of DNA-PK with NU7026 in LN-229 cells treated with TMZ, irrespective of Rad51 knockdown, had no significant effect on survival (Fig. 8B), indicating that NHEJ does not protect significantly against TMZ. This is in line with our previous data obtained with rodent mutant cells, which showed that NHEJ only contribute marginally to the protection against O^6^-methylating agents [17]. Interestingly, DNA-PK inhibition had a significant sensitization effect on LN-229 glioma cells treated with γ-rays (Fig. 8B), which supports the observation that NHEJ was indeed inhibited in these cells. The results suggest a divergent role for this pathway in the repair of TMZ and ionizing radiation-induced DNA damage.

Synthetic lethality caused by PARP inhibitors in HR-deficient cells [24] has paved the way for several clinical trials for the use of these inhibitors in HR-deficient tumors. This prompted us to study the effect of PARP inhibition on Rad51 knockdown cells. As shown in Fig. 8C, PARP inhibition with olaparib resulted in increased killing in glioma cells knocked-down for Rad51 compared to controls. Co-treatment with TMZ and olaparib increased cell kill in both control and knocked-down cells; the most drastic decrease in survival was observed in the latter. The data indicates that glioma cells are unable to deal with TMZ-induced DNA damage when PARP is inhibited, which is exacerbated when HR is down-regulated by Rad51 knockdown.

### Outlook

Collectively, the data shows a dramatic increase in the sensitivity of glioma cells treated with O^6^-methylating (TMZ) and O^6^-chlorethylating (ACNU) anticancer drugs once HR was impaired by knockdown of its major players Rad51 and BRCA2. This was demonstrated in glioma cell lines proficient (LN-229 and U87MG) and deficient (T98G) for p53. Although MGMT completely abolished the glioma cell sensitization achieved by HR knockdown, its pharmacological inhibition by the pseudosubstrate O^6^BG restored it. The data also corroborates previous observations indicating a minor role of NHEJ in the repair of O^6^MeG triggered DSBs [17], and indicates a potential use of PARP inhibitors to further enhance TMZ-induced toxicity in glioma cells. As O^6^AA are employed so widely as part of the treatment of different tumors, especially in the metastatic state and when complete surgical removal of the neoplasm is not possible, enhancement of chemotherapy efficacy is highly desired.

While we demonstrated that down-regulation of Rad51 and BRCA2 greatly sensitizes glioma cells to TMZ and ACNU and, therefore, are potential new targets in glioma therapy, the limitation of this approach should also be pondered. High MGMT levels still provoke resistance and therefore tumors with an unfavorable MGMT status will still represent a major hurdle. We should note, however, that HR also protects against N-alkylations (Nikolova et al., in preparation), which are formed by TMZ and CNUs at high amounts and are repaired by base excision repair. It is thus conceivable that inhibition of repair of N-alkylations together with knockdown of HR might be a strategy for targeting tumors that express high MGMT levels.

Another important issue pertains to the tumor targeting for HR knockdown strategies, in order to prevent unwanted side effects due to enhanced normal tissue toxicity. Indeed, systemic down-regulation of HR might exacerbate the side effects related to chemotherapeutics. Therefore, either local administration directly to the tumor or a proper tumor targeting strategy for iRNA [25], [26] and MGMT inhibitors [5], [27] might be fundamental for optimizing the therapeutic index of this approach. In summary, the study provides proof of principle evidence that siRNA targeting the HR pathway is a reasonable strategy for increasing O^6^AA efficacy, which may prove beneficial for patients treated with these anticancer drugs.

## Materials and Methods

### Cell lines and culture conditions

LN-229, U87MG and T98G cells were provided by Dr. M. Weller (Department of Oncology, University Hospital Zurich, Switzerland) [28] and were checked before experimental use for mycoplasma contamination. Cells were cultured in Dulbecco's modified Eagle's medium (DMEM) containing 10% fetal bovine serum as previously described [29], [30]. Cells were cultured in a humidified atmosphere with 7% CO_2_ at 37°C.

### Drugs, treatments and irradiation of cells

TMZ (a generous gift from Schering-Plough) stocks were prepared by dissolving it in DMSO and sterile dH_2_O (1∶2) to a concentration of 35 mM. ACNU (Sigma) stocks were prepared by dissolving it in sterile dH_2_O to 10 mM. O^6^-benzylguanine (O^6^BG, Sigma), NU7026 (Sigma) and Olaparib (AZD2281, Selleck Chemicals) stocks were prepared by dissolving them in DMSO to 10 mM, 5 mM and 10 mM, respectively. TMZ stocks were stored at −80°C while the other stocks were stored at −20°C. For MGMT depletion or PARP inhibition, O^6^BG (10 µM) or Olaparib was added 1 h prior to drug treatments. NU7026 (10 µM) was added to cells 6h prior to treatments. Radiation was performed with a ^137^Cs source (Gammacell 2000, Molsgaard Medical).

### Plasmids and stable transfections

A pSuper (OligoEngine) construct was generated to express shRNA targeting Rad51 mRNA using the previously described sequence (5′- GAAGAAAUUGGAAGAAGCU-3′) [31]. The pSV2MGMT vector has been described previously [32]. Plasmid DNA were transfected using Effectene (Qiagen). Transfected cells were selected with 0.75 mg/ml G418 or 0.44 µg/ml puromycin (both from Invitrogen) until clones formed.

### Transient transfection

BRCA2 siRNA (5′-CUGAGCAAGCCUCAGUCAAtt-3′), Rad51 siRNA (5̀-GAAGAAAUUGGAAGAAGCUtt-3′), (target sequences previously reported in [33], and [31], respectively) or non-sense siRNA (AllStars Negative Control siRNA, Qiagen) were transfected with Lipofectamine RNAiMAX (Invitrogen).

### Flow cytometric analysis

For Sub-G1 analysis, harvested cells were fixed in 70% ethanol at -20°C. RNA digested-cells (30 µg/ml RNase A for 1 h in PBS) were stained with 16.7 µg/ml propidium iodide (PI) prior to FACS analysis. Annexin V-FITC/ PI double-staining was performed following the manufacturer specifications (BD Pharmingen). Flow cytometry analysis was performed using a FACSCalibur (Becton Dickinson).

### Colony Formation Assay

Cells in logarithmic growth were seeded at appropriate numbers on 60 mm dishes. Cells were allowed to attach for 6 h before irradiation or drug treatments. Colonies were fixed in acetic acid∶methanol∶H_2_O (1∶1∶8), and stained with 1.25% Giemsa and 0.125% violet crystal. Transient transfected cells were seeded on dishes 18 h after transfection.

### Immunofluorescence microscopy

Cells were sequentially fixed with 4% formaldehyde and 100% methanol. Primary antibodies (anti-phospho-S139-H2AX, 1∶1000, Upstate; anti-53BP1, 1∶400, Cell Signaling) were incubated overnight at 4°C followed by 2 h incubation with the secondary antibody (anti mouse- Alexa Fluor 488, 1∶500, Invitrogen; anti-rabbit-Cy™3, 1∶500, Jackson ImmunoResearch Laboratories). DNA was counterstained either with 100 nM DAPI or 1 µM TO-PRO-3 (Invitrogen). Slides were mounted in 1% DABCO- 50% glycerol- PBS. Foci were scored automatically with the Metafer Finder System v.3.1 (MetaSystems). Microphotographs were acquired by laser scanning microscopy (LSM710, Carl Zeiss MicroImaging).

### DNA-PK activity assay

Total cell extracts were prepared as described [34]. Briefly, cells were lysed using three cycles of freeze (liquid nitrogen)/thaw (30°C) in extraction buffer (50 mM NaF, 20 mM HEPES (pH 7.8), 450 mM NaCl, 25% glycerol, 0.2 mM EDTA, 0.5 mM dithiothreitol, in the presence of protease inhibitors (Complete. EDTA-free. Roche)). Extracts were centrifuged (12 000 rpm for 30 min at 4°C), and supernatants were shock frozen and stored at −80°C. Endogenous DNA was removed by DEAE Sepharose Fast Flow (GE Healthcare). DNA-PK activity was determined by liquid scintillation counting using the SignaTECT DNA-Dependent Protein Kinase Assay System (Promega). The assay was performed on three independent cell extracts for each cell line. Each sample was analyzed in the presence or absence of 10 µM of the DNA-PKcs inhibitor NU7026. For background control, reactions were performed in the absence of activator, as suggested by the assay manufacturer.

### Western blot analysis

Samples were resolved by SDS-PAGE, and blotted onto a nitrocellulose membrane. Primary antibodies [BRCA2 (Cell Signaling), Erk-2 (Santa Cruz), MGMT (Millipore), Rad51 (Calbiochem)] were used and detected by the Odyssey® Infrared Imaging System (LI-COR Biotechnology).

## References

[1] Stupp R, Mason WP, van den Bent MJ, Weller M, Fisher B (2005). Radiotherapy plus concomitant and adjuvant temozolomide for glioblastoma.. N Engl J Med.

[2] Kaina B, Christmann M, Naumann S, Roos WP (2007). MGMT: key node in the battle against genotoxicity, carcinogenicity and apoptosis induced by alkylating agents.. DNA Repair (Amst).

[3] Hegi ME, Diserens AC, Gorlia T, Hamou MF, de Tribolet N (2005). MGMT gene silencing and benefit from temozolomide in glioblastoma.. N Engl J Med.

[4] Christmann M, Nagel G, Horn S, Krahn U, Wiewrodt D (2010). MGMT activity, promoter methylation and immunohistochemistry of pretreatment and recurrent malignant gliomas: a comparative study on astrocytoma and glioblastoma.. Int J Cancer.

[5] Kaina B, Margison GP, Christmann M (2010). Targeting O-methylguanine-DNA methyltransferase with specific inhibitors as a strategy in cancer therapy.. Cell Mol Life Sci.

[6] Karran P, Bignami M (1994). DNA damage tolerance, mismatch repair and genome instability.. Bioessays.

[7] Ochs K, Kaina B (2000). Apoptosis induced by DNA damage O6-methylguanine is Bcl-2 and caspase-9/3 regulated and Fas/caspase-8 independent.. Cancer Res.

[8] Mojas N, Lopes M, Jiricny J (2007). Mismatch repair-dependent processing of methylation damage gives rise to persistent single-stranded gaps in newly replicated DNA.. Genes Dev.

[9] Quiros S, Roos WP, Kaina B (2010). Processing of O6-methylguanine into DNA double-strand breaks requires two rounds of replication whereas apoptosis is also induced in subsequent cell cycles.. Cell Cycle.

[10] Roos WP, Batista LF, Naumann SC, Wick W, Weller M (2007). Apoptosis in malignant glioma cells triggered by the temozolomide-induced DNA lesion O6-methylguanine.. Oncogene.

[11] Weterings E, Chen DJ (2008). The endless tale of non-homologous end-joining.. Cell Res.

[12] Lieber MR (2010). The mechanism of double-strand DNA break repair by the nonhomologous DNA end-joining pathway.. Annu Rev Biochem.

[13] San Filippo J, Sung P, Klein H (2008). Mechanism of eukaryotic homologous recombination.. Annu Rev Biochem.

[14] Li X, Heyer WD (2008). Homologous recombination in DNA repair and DNA damage tolerance.. Cell Res.

[15] Jensen RB, Carreira A, Kowalczykowski SC (2010). Purified human BRCA2 stimulates RAD51-mediated recombination.. Nature.

[16] Liu J, Doty T, Gibson B, Heyer WD (2010). Human BRCA2 protein promotes RAD51 filament formation on RPA-covered single-stranded DNA.. Nat Struct Mol Biol.

[17] Roos WP, Nikolova T, Quiros S, Naumann SC, Kiedron O (2009). Brca2/Xrcc2 dependent HR, but not NHEJ, is required for protection against O(6)-methylguanine triggered apoptosis, DSBs and chromosomal aberrations by a process leading to SCEs.. DNA Repair (Amst).

[18] Beranek DT (1990). Distribution of methyl and ethyl adducts following alkylation with monofunctional alkylating agents.. Mutat Res.

[19] Nikolova T, Ensminger M, Lobrich M, Kaina B (2010). Homologous recombination protects mammalian cells from replication-associated DNA double-strand breaks arising in response to methyl methanesulfonate.. DNA Repair (Amst).

[20] Citron M, White A, Decker R, Wasserman P, Li B (1995). O6-methylguanine-DNA methyltransferase in human brain tumors detected by activity assay and monoclonal antibodies.. Oncol Res.

[21] Rogakou EP, Pilch DR, Orr AH, Ivanova VS, Bonner WM (1998). DNA double-stranded breaks induce histone H2AX phosphorylation on serine 139.. J Biol Chem.

[22] Rodrigue A, Lafrance M, Gauthier MC, McDonald D, Hendzel M (2006). Interplay between human DNA repair proteins at a unique double-strand break in vivo.. EMBO J.

[23] Willmore E, de Caux S, Sunter NJ, Tilby MJ, Jackson GH (2004). A novel DNA-dependent protein kinase inhibitor, NU7026, potentiates the cytotoxicity of topoisomerase II poisons used in the treatment of leukemia.. Blood.

[24] Bryant HE, Schultz N, Thomas HD, Parker KM, Flower D (2005). Specific killing of BRCA2-deficient tumours with inhibitors of poly(ADP-ribose) polymerase.. Nature.

[25] Boudreau RL, Davidson BL (2010). RNAi therapeutics for CNS disorders.. Brain Res.

[26] Bowers WJ, Breakefield XO, Sena-Esteves M (2011). Genetic therapy for the nervous system.. Hum Mol Genet.

[27] Koch D, Hundsberger T, Boor S, Kaina B (2007). Local intracerebral administration of O(6)-benzylguanine combined with systemic chemotherapy with temozolomide of a patient suffering from a recurrent glioblastoma.. J Neurooncol.

[28] Weller M, Rieger J, Grimmel C, Van Meir EG, De Tribolet N (1998). Predicting chemoresistance in human malignant glioma cells: the role of molecular genetic analyses.. Int J Cancer.

[29] Batista LF, Roos WP, Christmann M, Menck CF, Kaina B (2007). Differential sensitivity of malignant glioma cells to methylating and chloroethylating anticancer drugs: p53 determines the switch by regulating xpc, ddb2, and DNA double-strand breaks.. Cancer Res.

[30] Hermisson M, Klumpp A, Wick W, Wischhusen J, Nagel G (2006). O6-methylguanine DNA methyltransferase and p53 status predict temozolomide sensitivity in human malignant glioma cells.. J Neurochem.

[31] Biard DS (2007). Untangling the relationships between DNA repair pathways by silencing more than 20 DNA repair genes in human stable clones.. Nucleic Acids Res.

[32] Kaina B, Fritz G, Mitra S, Coquerelle T (1991). Transfection and expression of human O6-methylguanine-DNA methyltransferase (MGMT) cDNA in Chinese hamster cells: the role of MGMT in protection against the genotoxic effects of alkylating agents.. Carcinogenesis.

[33] Fan S, Meng Q, Auborn K, Carter T, Rosen EM (2006). BRCA1 and BRCA2 as molecular targets for phytochemicals indole-3-carbinol and genistein in breast and prostate cancer cells.. Br J Cancer.

[34] Shao CJ, Fu J, Shi HL, Mu YG, Chen ZP (2008). Activities of DNA-PK and Ku86, but not Ku70, may predict sensitivity to cisplatin in human gliomas.. J Neurooncol.

